# Transcriptomic Effects of Healthspan-Promoting Dietary Interventions: Current Evidence and Future Directions

**DOI:** 10.3389/fnut.2021.712129

**Published:** 2021-08-10

**Authors:** Devin Wahl, Thomas J. LaRocca

**Affiliations:** ^1^Department of Health and Exercise Science, Colorado State University, Fort Collins, CO, United States; ^2^Center for Healthy Aging, Colorado State University, Fort Collins, CO, United States

**Keywords:** aging, RNA-Seq - RNA sequencing, transcriptome, healthspan, nutrition

## Abstract

Aging is the greatest risk factor most diseases, including cardiovascular disorders, cancers, diabetes, and neurodegeneration, but select nutritional interventions may profoundly reduce the risk for these conditions. These interventions include calorie restriction, intermittent fasting, protein restriction, and reducing intake of certain amino acids. Certain *ad libitum* diets, including the Mediterranean, Finnish Geriatric Intervention Study to Prevent Cognitive Impairment and Disability, and Okinawan diets also promote healthy aging. Evidence indicates that these dietary strategies influence aging and healthspan by acting on the biological “hallmarks of aging” and especially upstream nutrient sensing pathways. Recent advances in “omics” technologies, including RNA-sequencing (transcriptomics), have increased our understanding of how such nutritional interventions may influence gene expression related to these biological mediators of aging, primarily in pre-clinical studies. However, whether these effects are also reflected in the human transcriptome, which may provide insight on other downstream/related cellular processes with aging, is an emerging topic. Broadly, the investigation of how these nutritional interventions influence the transcriptome may provide novel insight into pathways associated with aging, and potential targets to treat age-associated disease and increase healthspan. Therefore, the purpose of this mini review is to summarize what is known about the transcriptomic effects of key dietary/nutritional interventions in both pre-clinical models and humans, address gaps in the literature, and provide insight into future research directions.

## Introduction

Older age is the major risk factor for cardiovascular disorders, cancers, diabetes, sarcopenia, frailty, and neurodegeneration (1). As a result, the “Geroscience” concept has emerged in an attempt to understand the relationships between aging biology and age-related diseases, with the hope that targeting aging itself will increase healthspan (the number of healthy, disease-free years in older age) (2). The mechanisms that drive aging/age-related diseases, termed the “hallmarks of aging,” represent key molecular targets for interventions to improve healthspan (3).

Nutrition has a profound impact on the biology of aging and disease. Several interventions in particular act on most hallmarks of aging to improve cardiometabolic, physical and cognitive health, and to delay age-related disease and increase lifespan. These interventions include calorie restriction (CR), intermittent fasting (IF) (4), alternate day fasting (ADF), prolonged fasting (PF), time-restricted feeding (TRF), protein restriction, and reduced intake of certain amino acids. Specific eating patterns, including the Mediterranean, Okinawan, and FINGER (Finnish Geriatric Intervention Study to Prevent Cognitive Impairment and Disability) diets also improve health (5). Information on how these interventions impact health and lifespan (largely by influencing the hallmarks of aging) has accumulated over several decades (1). However, recent advances in “omics” technologies, including RNA-sequencing, may provide additional, important understanding of the common or distinct biological mechanisms underlying these interventions and yield novel insight into targetable, genomic/cellular pathways associated with aging and disease.

**Figure 1:**
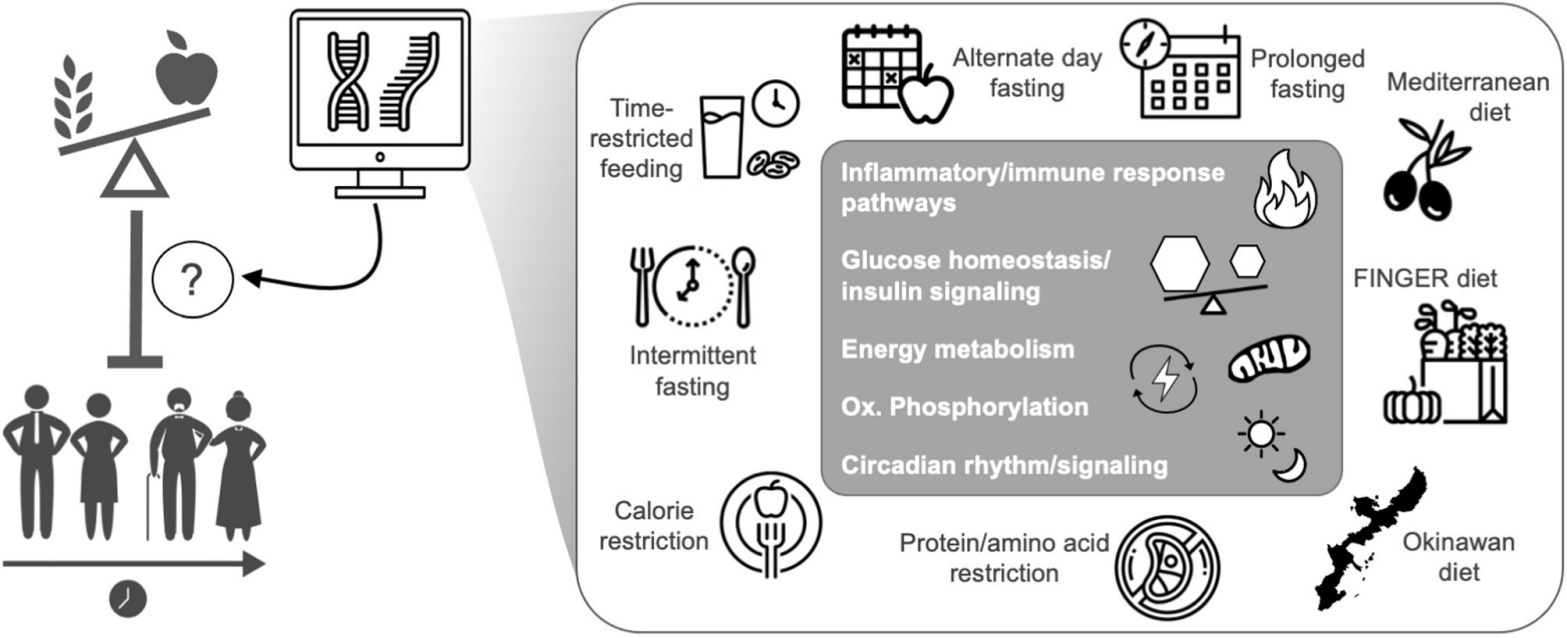
Transcriptomics has increased the understanding of how health-promoting nutritional interventions influence gene expression related to the biological mediators of aging and healthspan, mostly in pre-clinical models. The transcriptomic effects of healthspan-promoting nutritional interventions are still under investigation, but common effects among all interventions are noted in the gray box. Icons from nounproject.com under Creative Commons license.

The effects of healthy aging interventions on the transcriptome are not fully understood, mostly due to a lack of sequencing data. However, evidence to date suggests that key transcriptomic pathways involved in aging including glucose homeostasis/insulin signaling, inflammation, oxidative phosphorylation, immune responses, and circadian signaling are similarly influenced by most healthy aging/nutritional interventions (Figure 1 and Table 1). The purpose of this mini review is to detail how healthy aging interventions affect these pathways (and related genes/modulators), and to outline additional pathways/genes that are distinctly modulated with select healthy dietary interventions. While most existing transcriptomic and genetic studies have used pre-clinical models, we also provide insight into how some of these interventions affect the human transcriptome. Finally, we describe gaps in the research, future directions, and goals in the field of dietary interventions and transcriptomics.

**Table 1:** Commonalities among the effects of select healthy dietary interventions on biological hallmarks of aging in transcriptomic evidence.

**Dietary intervention**	**Aging hallmark/transcriptomic pathways influenced by intervention**	**Species**	**Tissue(s)**	**Central signaling mediators**	**References**
Calorie restriction	Glucose dysregulation/insulin signaling **⇓**; Inflammation **⇓**; Oxidative phosphorylation **⇓**; Immune response **⇑⇓**; Circadian rhythm **⇑⇓**	Rodents, non-human primates, humans	Liver, muscle, brain, white adipose tissue, blood mononuclear cells	*FOXO1, FOX03, IGF-1, GH, MTOR (*insulin signaling*)* *IL-6, MIF, p53, NF-κB* (inflammation) *AMPK, SIRT1, PPARα, PGC1-*α (oxidative phosphorylation) C4, *C1qa, Lzp-s, NF-κB* (immunity) *PER1, PER2, CRY, ROR-α, ROR γ* (circadian rhythm)	(6–13)
Intermittent fasting	Glucose insulin signaling **⇓**; Inflammation **⇓**; Oxidative phosphorylation **⇓** Immune response **⇑⇓**; Circadian rhythm **⇑⇓**.	Rodents, humans	Liver, white adipose tissue, brain, muscle, blood mononuclear cells	*SIRT1, SIRT3, IGF-1, FOXO1, GH, MTOR, AMPK* (insulin signaling) *TNF-*α*, IL1B, IL-6* (inflammation) *PPARα, SIRT1, SIRT2, PGC1-*α (oxidative phosphorylation) *APOA2, HPX, NF-κB* (immune response) *PER1, PER2, CRY* (circadian rhythm)	(6, 14–19)
Alternate day fasting/pro-longed fasting	Glucose insulin signaling **⇓**; Inflammation **⇓**; Immune response **⇑⇓;** Circadian rhythm **⇑⇓**.	Rodents, some evidence in humans	Liver, brain, muscle, white adipose tissue	*SIRT1, SIRT3, IGF-1, FOXO1, GH, MTOR, AMPK* (insulin signaling) *TNF-α, IL-6, IL-1β, NF-κB* (inflammation) *NF-κB* (immune response) *PER 1, PER2, BMAL1 and CRY1, CRY2* (circadian rhythm)	(9, 19, 20)
Time restricted feeding	Glucose insulin signaling **⇓**; Inflammation **⇓**; Oxidative phosphorylation**⇓;** Immune response **⇑⇓**; Circadian rhythm **⇑⇓**.	Rodents, some evidence in humans	Liver, muscle, white adipose tissue	*SIRT1 SIRT3, AMPK, IGF-1, GH, MTOR* (insulin signaling) *TNF-α, IL-6, IL-1β* (inflammation) PPAR-α, SIRT1, SIRT3, AMPK (oxidative phosphorylation) *NF-κB* (immune response) *PER 1, PER2, BMAL1 and CRY1, CRY2* (circadian rhythm)	(9, 19–21)
Protein/amino acid restriction	Insulin signaling **⇓** Circadian rhythm **⇑⇓**; Immune response **⇓**	Rodents, some evidence in humans	Liver, kidney	*MTOR, AMPK, SIRT1* (insulin signaling) *NF-κB* (immune response) IL-6, IL-10, TNF*-*α (inflammation) *BMAL1, CRY1, NPAS2, REV-ERBα* (circadian rythym)	(10, 22, 23)

*Arrows indicate effects on the pathway (increased/decreased). Species, tissues, select central signaling mediators, and references are included*.

## Calorie Restriction

Perhaps the most powerful anti-aging nutritional intervention, CR is defined as a reduction in 10–50% daily caloric intake *without malnutrition*. CR increases healthspan and lifespan through many mechanisms, including improved glucose homeostasis and mitochondrial health, and by inhibiting all molecular hallmarks of aging (24). The transcriptomic effects of CR in pre-clinical models have been extensively investigated in multiple tissues. Seminal studies showed that long-term CR reverses gene expression changes associated with age-related inflammation and impaired stress responses, DNA-replication/cell cycle defects, oxidative stress, tumorigenesis, and macromolecular damage (11, 25, 26). Additional results indicate that CR largely reduces transcriptomic signatures associated with immune activation, inflammatory signaling, glucose homeostasis, and AMP-activated protein-kinase/insulin-like growth factor 1 (AMPK/IGF-1) signaling (evolutionarily conserved energy sensing pathways). Also, consistent with the observation that glucose homeostasis/insulin signaling are central players in the effects of CR on metabolism, CR changes the expression of key genes affecting energy/metabolism-modulating proteins and insulin secretion (largely reducing the expression of these genes) (6). In addition to glucose homeostasis, other pathways/genes contributing to DNA repair, fatty acid metabolism, and citric acid cycle/oxidative phosphorylation appear to be key transcriptomic mediators of the effects of CR in white adipose tissue and liver in pre-clinical models (27–29).

More recently, the influence of CR on the liver transcriptome in the *Rhesus macaque* (a close relative to humans sharing 93% DNA homology) was investigated. A two-year CR intervention reduced transcriptome signatures of immune activation and inflammation (a main hallmark of aging) and increased pathways associated with ribosome activity, branched-chain amino acid metabolism, fatty acid degradation, and RNA transport mechanisms (7). These results suggest that *Rhesus macaques* and mice respond similarly to CR, with main transcriptomic changes in inflammatory pathways, immune function, and fatty acid degradation (related to glucose metabolism) in multiple tissues (30).

In humans, analyses from multiple studies indicates that both short-term (e.g., 4 weeks) and long-term (e.g., 1 year) CR upregulates transcriptome modules associated with stem cell maintenance, blood vessel remodeling, and lipid metabolism in subcutaneous fat tissue (31). Other studies indicate that very long-term (10 years) voluntary CR causes gene expression patterns related to enhanced lipid and glucose metabolism, and downregulation of the insulin/IGF-1 growth pathway (resulting in a younger transcriptional profile) in muscle (a main metabolic tissue), consistent with the pre-clinical mouse and non-human primate data (8).

## Intermittent Fasting

IF is an umbrella term for eating patterns (which also include ADF, PF, and TRF) involving an extended time with little or no energy intake (fasting), all of which are associated with improved glucose homeostasis (and reduced insulin) and increased stress resistance (32). It has been suggested that the key benefit of IF comes from fasting periods (rather than reduced total energy intake), which can induce hormesis (responses to moderate stress with downstream protective and anti-aging effects). For example, 11 h/day of restricted feeding (e.g., no calorie intake) reduces liver pathology and extends median lifespan in male mice (33). IF (16 h/day) in mice also results in global transcriptomic changes in the liver (a key metabolic organ) including modulation of pathways contributing to sterol, alcohol, and cholesterol synthesis, and changes in gene regulation modulating circadian rhythm (14). In addition to these effects, IF enhances transcriptional signatures of peroxisome proliferator activator receptor (PPAR) activity, a major mediator of oxidative phosphorylation, highlighting the influence of IF on homeostasis and metabolic function (34). Finally, IF (12–16 h/day) results in global transcriptomic changes in the mouse brain reflecting increased neuroplasticity and reduced inflammation, which parallels the effects of CR on the brain (15).

The transcriptomic effects of IF in humans are still under investigation (studies are underway), but initial results from studies of individual genes indicate that IF (5 days of periodic fasting) increases transcriptomic signatures of nutrient and energy sensing/oxidative phosphorylation gene expression including AMP-activated protein kinase (AMPK), Forkhead box protein 01 (FOXO1), Sirtuin 1 (SIRT1), and Sirtuin 3 (SIRT 3). These genes/proteins are key players in cellular regulation pathways and centrally implicated stress responses, cardiometabolic/systemic health, and longevity (35). Another study (36) confirmed these results with data showing that IF contributes to a marginal increase in SIRT3 gene expression, which was associated with a decrease in plasma insulin levels. While these preliminary results in humans have primarily focused on changes in specific gene expression, the global transcriptomic effects of IF (36-h fast) appear to be similar to CR with changes in gene expression related to fatty acid oxidation, cell cycle and apoptosis pathways, and reductions in key inflammatory genes/pathways (16).

## Alternate Day Fasting and Prolonged Fasting

ADF and PF are types of IF involving feeding days (on which food is consumed *ad libitum*), and fasting days on which no calories are consumed. The fasting days can occur every other day or intermittently (e.g., fasting 2 days per week or even up to 21 days once per year, which is more commonly known as PF) (37, 38). Evidence shows that these dietary interventions induce metabolic effects similar to IF, including improving glucose homeostasis and reducing liver triglyceride content in mice (39). In pre-clinical models, these diets flip a “metabolic switch” activating key transcriptional regulators of fatty acid and energy metabolism and inflammation (9, 40), and transcriptional evidence shows that ADF (24-h fast) modulates key processes associated with circadian rhythm, RNA processing, and oxidative metabolism (41).

In humans, a prolonged 10-day fast results in transcriptomic signatures of increased lipolysis/lipid metabolism and reduced activation of pathways related to glycolysis and oxidative phosphorylation. Interestingly, this prolonged fasting period also contributes to the upregulation of inflammatory pathways and macrophage activation in subcutaneous fat tissue (42), reflecting a potential hormesis response and downstream protective mechanisms. Another study showed that short-term fasting is associated with enrichment of transcriptomic pathways involved in fatty acid oxidation, cell cycling and apoptosis, and a decrease in the expression of pro-inflammatory genes in peripheral blood mononuclear cells (16). These apparently divergent results suggest that the protocol, length of fasting, and tissues analyzed are important factors to consider when studying the influence of these dietary interventions on the human transcriptome.

## Time-Restricted Feeding

TRF is a another type of IF and allows for eating only during a certain time window in the day (e.g., eating *ad libitum* for 8 h or less a day in humans) (37). TRF is different from IF in that the daily eating time frame remains constant, which is thought to influence circadian clock pathways. TRF also promotes cardiometabolic/systemic health by maintaining metabolic homeostasis (e.g., improving glucose tolerance and insulin sensitivity, and reducing blood lipid levels) (43). Additional effects of TRF include enhanced mitochondrial health, DNA-repair, and autophagy, all of which coincide with improved glucose metabolism and potentially reduced risk for most age-related diseases (44). In mice, TRF contributes to transcriptomic changes to circadian clock gene expression (increased expression/rhythmicity) and metabolic/nutrient sensing pathways (changes that parallel improved metabolic health and the prevention of fatty liver) (21), all of which are similar to effects seen with IF, ADF, and PF.

There is limited research on the effects of TRF on the human transcriptome/gene expression, but recent evidence suggests that a daily 8-h eating time frame influences genes responsible for amino acid transport in muscle (45), and reduces gene expression signatures related to growth and metabolism. Research on the effects of fasting has recently focused on peripheral/circadian clock genes, with some studies suggesting no universal effect (17) and others suggesting that that TRF in particular influences several genes implicated in circadian rhythm (46). Such changes in circadian clock gene expression might impact healthspan and lifespan, but these links are poorly understood (47).

## Protein and Amino Acid Restriction

The restriction of dietary protein and certain amino acids promotes healthspan and lifespan in pre-clinical models (48) and may mimic CR to some extent. This may be an important observation, especially considering that most people may find it difficult to adhere to most calorie deficit interventions, especially in Western societies where there is essentially free access to calorie-dense foods. Long-term reduction of dietary protein intake (through dilution with non-digestible fiber) improves healthspan and lifespan via reduced growth pathway activation and improved mitochondrial function in male and female mice (49), and short-term protein restriction reduces circulating triglycerides via changes in hepatic APOE expression (50). Similarly, reduced intake of branched-chain amino acids (BCAAs; leucine, isoleucine, and valine) also improves health and lifespan in male and female mice by inhibiting mechanistic target of rapamycin (MTOR) nutrient sensing pathway activation, improving glucose homeostasis, and reducing insulin resistance (22). While the aforementioned studies are not transcriptomic studies, the results suggest that protein and amino acid restriction may partly mimic the gene expression changes and metabolic effects seen with fasting or related nutritional interventions.

Protein restriction is known influence key genes associated with circadian rhythm, metabolic signaling (including insulin signaling), and oxidative phosphorylation, in addition to other transcriptional factors/genes linked with healthspan and lifespan extension (51, 52). On the other hand, BCAA intake is associated with transcriptomic effects including changes in cell cycle/metabolic pathways, apoptosis (programmed cell death), p53 activity (cell division), and NF-κB signaling (innate and adaptive immunity) (22). These results highlight the contribution of these diet-induced transcriptomic effects to changes in metabolic/energy-sensing processes, as well as the modulation of inflammation—all of which are associated with aging and age-related disease (53).

## Common Transcriptomic Pathways Affected by Most Well-Studied Healthy Dietary Interventions

The common transcriptomic pathways modulated by most health-promoting dietary interventions involve glucose/insulin signaling, inflammation, oxidative phosphorylation, immune responses, and circadian rhythm (Table 1). Based on limited transcriptomic/genetic data, these processes seem to be similarly affected by most interventions, regardless of species or tissue, although specific genes within pathways may be activated/repressed depending on the intervention type (e.g., the duration of CR or fasting period). We also note that some processes like circadian rhythm and immunity are especially complex and may be differentially modulated (e.g., increased or decreased) and highly dependent on length or type of intervention (54). Below, we briefly describe the common biological processes/transcriptomic pathways that are affected by most healthy aging interventions.

### Glucose Homeostasis and Insulin Signaling

Metabolic dysfunction is considered a central hallmark of aging, and current transcriptomic data shows that all healthy aging dietary interventions influence pathways involved with glucose homeostasis and insulin signaling. Most evidence suggests that these interventions improve health by suppressing key insulin-related signaling pathways and gene expression. These main genes include Insulin-like growth factor-1 (IGF-1, reduced), Sirtuins (SIRT1 and SIRT3, increased), Growth hormone (GH, increased), Mechanistic target of Rapamycin (MTOR, reduced), and FOXO genes (increased) (6), all of which have key roles in energy sensing, growth and metabolism (5).

### Inflammation

Inflammation is considered a key hallmark of aging because it can directly affect all other biological hallmarks of aging (55). Most healthy dietary interventions targeting energy intake reduce transcriptomic evidence of inflammatory activation, and this has been demonstrated in multiple species/tissues. Key inflammatory mediators involved in these pathways include Interluekin-6 (IL-6), Tumor necrosis factor alpha (TNF-α), and Interleukin 1 beta (IL-1β). Importantly, additional health-promoting diets including the FINGER and Mediterranean (described below) also similarly modulate these pro-inflammatory cytokines (and transcriptomic evidence of their activity) in humans (56).

### Immune Response

Long periods of calorie restriction or periodic fasting may impair immune system pathways, while short bouts of fasting are emerging as potential interventions for immune system repair and maintenance (10). There is evidence that most healthy aging interventions influence immune responses to a certain degree, mostly by modulating *NF-*κ*B*, a transcriptional factor which plays critical roles in immune response and cytokine production (38). Some evidence suggests that CR reduces additional transcripts (57) involved in immune function including Complement component 4 (*C4*) and Complement C1q (*C1q*), but whether these genes are also influenced by the other interventions remains to be determined.

### Oxidative Phosphorylation

All healthy nutritional interventions act on the mitochondria, and consequently, pathways/genes contributing to cellular respiration. Most evidence suggests that these interventions reduce oxidative phosphorylation gene expression (58) consequently attenuating the production of reactive oxygen species (ROS; a key contributor to aging/age-related disease). Increased expression/activity of mitochondrial biogenesis and turnover pathways with healthy nutritional interventions may also contribute to greater mitochondrial efficiency (despite reductions in oxidative phosphorylation). Key genes associated with these pathways are mostly energy sensing in nature and include the Sirtuins, Peroxisome proliferator activated-receptor α (PPAR-α), and AMPK (59).

### Circadian Rhythm

Most physiological and metabolic processes are regulated by circadian clock genes that are responsible for the synchronization of biological processes within an organism (60). Recently, it has been suggested that circadian rhythm dictated by peripheral clock genes can be re-synchronized (thus resulting in positive health benefits) under CR or PF (61). Key modulators of circadian rhythm pathways affected by most nutritional interventions include the Period circadian regulators (PER1 and PER2) and the Cryptochromes (CRY) (61).

## Exploration of the Transcriptomic Effects of Additional Health-Promoting Diets

Most of the well-studied interventions above are calorie or macronutrient restrictive. It is therefore important to understand how other health-promoting *ad libitum* nutritional interventions influence the genome. Indeed, emerging data show that several healthy dietary/nutritional interventions or patterns act on the transcriptome to affect processes associated with aging, age-related disease, and longevity.

The Finnish Geriatric Intervention Study to Prevent Cognitive Impairment and Disability (FINGER), Mediterranean, and Okinawan diets all improve health in humans. These diets are characterized by ample fruit and vegetable consumption and low-to-moderate protein intake (with limited red meat consumption), low-to-moderate fat consumption (healthy fats including olive oils and avocado), and moderate-to-high carbohydrate consumption (whole grains, potatoes) (62, 63). The FINGER diet also includes cognitive function-enhancing exercises and a weekly physical activity routine (64).

The most well-studied of these diets, in terms of transcriptomic impact, is the Mediterranean diet. The Mediterranean diet elicits a transcriptomic response in humans including reduced inflammatory/inflammation response gene expression (65) (possibly due to a high consumption of monounsaturated fatty acids and olive oils) and enhanced circadian clock transcript levels (66). Other studies show that the Mediterranean diet affects transcriptomic pathways involved with cardiovascular health (e.g., atherosclerosis and hypertension) including lipid and cholesterol metabolism, which appears to be a main benefit of this diet (67). The transcriptomic effects of an Okinawan or FINGER dietary intervention remain to be determined; however, based on the above observations that the Mediterranean diet may have transcriptomic effects similar to those of CR/fasting, profiling the influence of these other diets on the transcriptome could be critical for identifying the ideal, non-fasting dietary pattern to promote healthy aging.

## Research Gaps and Future Directions

**Figure 2:**
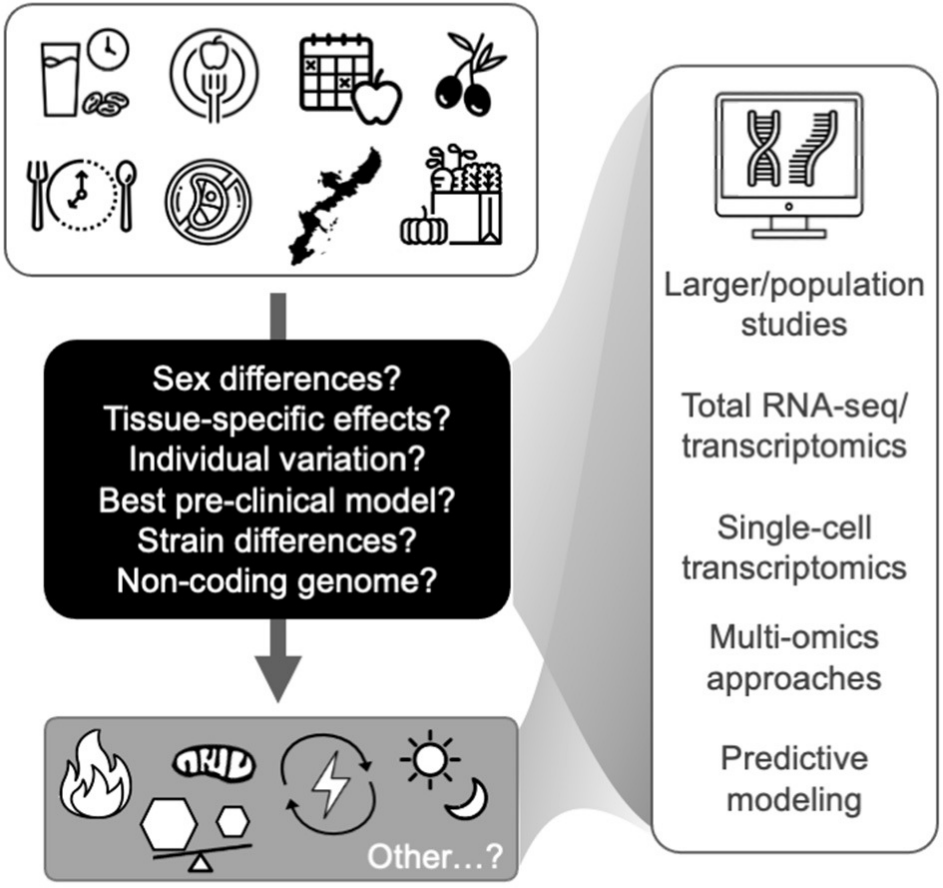
Future studies are needed to identify key transcriptomic modules that relate to the hallmarks of aging (bottom gray box) and represent targets for healthy aging in humans. Future directions and key issues (shown in the black box and at right) in transcriptomic studies of healthspan-promoting nutritional interventions are also noted. Icons from nounproject.com under Creative Commons license.

As described above and in Figure 1 and Table 1, the available evidence suggests that the common transcriptomic effects of healthspan-promoting nutritional interventions include favorable modulation of gene expression patterns related to inflammatory signaling, immune function, energy sensing/glucose homeostasis, oxidative phosphorylation (including mitochondrial function), and circadian regulation. Importantly, most of this evidence is based on: (1) pre-clinical studies with some limited/emerging clinical data; and (2) various experimental and methodological approaches, including gene expression/qPCR panels, microarrays, and most recently RNA-sequencing (see individual references for details). More research is needed to increase our understanding of human-specific transcriptomic effects of these interventions, and to leverage newer, comprehensive RNA-seq approaches—ideally in “multi-omics” studies (e.g., RNA-seq combined with proteomics) that may provide further mechanistic insight. In addition to these general future directions, below (and in Figure 2) we outline several key issues that require further investigation in this area.

### Suitability of Rodent Models

It is generally accepted that common responses to the most robust healthspan-promoting nutritional interventions are shared among mice and non-human primates, (68). However, it remains to be determined whether mice are a good translational model for studying the transcriptomic impact of nutritional interventions as they relate to humans. With CR (perhaps the most studied and robust nutritional intervention) for example, global plasma biomarker changes with CR in mice mimic those seen with fasting in humans (69), but recent research shows more divergent transcriptome effects of certain healthy nutritional interventions in mice and humans. For instance, an increase in stem cell maintenance and vascularization pathway activation is associated with CR in human subcutaneous white adipose tissue (WAT), but in mouse epididymal WAT these processes are downregulated with CR (31).

In addition to issues related to divergent mouse-human transcriptome effects, some commonly used mouse strains do not respond well to CR (e.g., they exhibit reduced lifespan) and/or other interventions. Most research on nutritional interventions and the transcriptome has utilized C57/B6 mice, a homogenous, inbred strain. However, It appears that CR results in greater lifespan extension in non-inbred mice when compared to inbred mice and may not be as effective in certain genetic backgrounds/strains (70). The underlying mechanisms are still under investigation, but some evidence shows that certain mouse strains exhibit completely opposite transcriptional responses to CR compared with other strains (e.g., BALBC, DBA), and these effects are tissue-specific (31). As such, when examining the influence of healthy nutritional interventions on the rodent transcriptome, it is important to consider the genetic strain/background and tissue(s) before drawing conclusions. This point parallels the need to consider genetic variability in humans (71) when considering the role and transcriptomic profiles of healthy aging interventions in aging/age-related disease. Indeed, it may be necessary to use transcriptomics and/or other omics approaches to develop “precision” dietary strategies tailored to individual people.

### Tissues for Studying the Effects of Nutritional Interventions on the Transcriptome

Most pre-clinical nutritional intervention studies have largely focused on transcriptomic changes in liver and fat, and to a lesser extent, in brain and muscle. Liver, fat, and muscle are key metabolic tissues that are centrally implicated in the transcriptomic and metabolic responses to healthy aging nutritional interventions (72), and it appears that the responses to certain dietary interventions are different depending on type of tissue analyzed. Some transcriptomic pathways, like the insulin/IGF1 signaling response, seem to be equally affected by fasting across multiple tissues in rodents (73). However, the major metabolic tissues/organs of the body have vastly different metabolic rates (74) and may therefore respond to nutritional interventions differently in other gene expression pathways. As an example, AMPK, a key energy sensor and metabolism regulator, is activated by fasting in metabolic tissues like fat, liver, and muscle. However, the activation/response of AMPK (and related downstream mechanisms) to fasting in other tissues (e.g., brain, kidney) may vary (75). Current and future research should aim to determine transcriptomic overlap and differences of healthy nutritional interventions in multiple tissues, as this will broaden the scope of targetable mechanisms in tissue-specific diseases.

### Sex-Specific Effects of Healthy Nutritional Interventions on the Aging Transcriptome

Male and female mice respond differently to anti-aging nutritional interventions (76). Key differences between sexes in response to nutritional interventions could be due to amino acid metabolism in the liver (77) or the ability of female mice to use increased energy from fat (through β-oxidation) during food restriction (78). Male and female mice also respond differently to varying intervention regimens (e.g., 20 vs. 40% CR) with different cardiometabolic and lifespan responses (24). Some recent RNA-seq studies have addressed these differences and found sex-specific responses in gene expression patterns with fasting and amino-acid restriction (79). Future research should continue to focus on such similarities/differences in transcriptomic responses to healthy aging nutritional interventions in both male and female mice, which may be key for understanding clinical applications.

### The Non-coding Genome and Healthy Aging Nutritional Interventions

The non-coding genome (e.g., non-coding RNAs, microRNAs) is often ignored in transcriptomic studies on aging. However, certain non-coding transcripts are emerging as key players in aging and disease, and could be critical in understanding the transcriptomic effects of nutritional interventions. For example, microRNAs, which negatively regulate gene expression of their targets at post-transcriptional levels, may be intricately involved in aging gene expression pathways and the hallmarks of aging (e.g., genome stability) (80). Some non-coding/microRNAs are influenced by CR to modify chromatin, which may partly explain how this intervention regulates gene expression during aging (81). Even less commonly investigated, non-coding repetitive elements (RE) make up more than 60% of the genome (82), and it has been suggested that some of these RE can become active during aging, perhaps due to decreased chromatin stability (83). Indeed, there is a global increase in RE transcripts in model organisms and humans with aging (84), and some of these RE may be associated with certain hallmarks of aging, including inflammation, oxidative stress, and cellular senescence (85, 86). Interestingly, CR is associated with a global reduction in RE transcripts in liver, which further supports the idea that global RE transcript dysregulation may be an important transcriptional hallmark of aging and healthy nutritional interventions (28). The influence of other nutritional interventions on RE with aging remains to be determined, but could be a promising area of future investigation.

## Conclusions

Nutrition has a profound impact on aging/age-related disease and lifespan. The transcriptomic effects of healthy nutritional interventions are still under investigation, but glucose regulation/insulin sensitivity (growth/metabolic homeostasis), oxidative phosphorylation, inflammatory/immune function, and circadian pathways appear to be key mediators of healthspan that are modulated by these interventions at the transcriptome level in multiple metabolic tissues and species. In order to identify additional genes/transcriptomic modules that may represent targets for promoting healthy aging, future transcriptome/multi-omic studies are needed to address key issues (e.g., differential sex and tissue responses, more comprehensive genomic profiling) and characterize the effects of more diets (e.g., Okinawan)—ideally in human subjects.

## Author Contributions

All authors listed have made a substantial, direct and intellectual contribution to the work, and approved it for publication.

## Conflict of Interest

The authors declare that the research was conducted in the absence of any commercial or financial relationships that could be construed as a potential conflict of interest.

## Publisher's Note

All claims expressed in this article are solely those of the authors and do not necessarily represent those of their affiliated organizations, or those of the publisher, the editors and the reviewers. Any product that may be evaluated in this article, or claim that may be made by its manufacturer, is not guaranteed or endorsed by the publisher.
